# The CSIRO Healthy Diet Score: An Online Survey to Estimate Compliance with the Australian Dietary Guidelines

**DOI:** 10.3390/nu9010047

**Published:** 2017-01-09

**Authors:** Gilly A. Hendrie, Danielle Baird, Rebecca K. Golley, Manny Noakes

**Affiliations:** 1 CSIRO Health and Biosecurity, P.O. Box 10041, Adelaide SA 5000, Australia; danielle.baird@csiro.au (D.B.); manny.noakes@csiro.au (M.N.); 2 School of Pharmacy and Medical Sciences, University of South, Adelaide SA 5000, Australia; rebecca.golley@unisa.edu.au

**Keywords:** dietary assessment, food survey, diet quality, dietary patterns, Australia, online

## Abstract

There are few dietary assessment tools that are scientifically developed and freely available online. The Commonwealth Scientific and Industrial Research Organisation (CSIRO) Healthy Diet Score survey asks questions about the quantity, quality, and variety of foods consumed. On completion, individuals receive a personalised Diet Score—reflecting their overall compliance with the Australian Dietary Guidelines. Over 145,000 Australians have completed the survey since it was launched in May 2015. The average Diet Score was 58.8 out of a possible 100 (SD = 12.9). Women scored higher than men; older adults higher than younger adults; and normal weight adults higher than obese adults. It was most common to receive feedback about discretionary foods (73.8% of the sample), followed by dairy foods (55.5%) and healthy fats (47.0%). Results suggest that Australians’ diets are not consistent with the recommendations in the guidelines. The combination of using technology and providing the tool free of charge has attracted a lot of traffic to the website, providing valuable insights into what Australians’ report to be eating. The use of technology has also enhanced the user experience, with individuals receiving immediate and personalised feedback. This survey tool will be useful to monitor population diet quality and understand the degree to Australians’ diets comply with dietary guidelines.

## 1. Introduction

Measuring dietary intake in large population groups can be difficult when time and resources are limited. Traditional methods of assessment may have a high participant burden, and also take time for researchers to manage the data entry and conduct the analysis. Adapting traditional methods to incorporate technology is one way researchers have tried to reduce the burden of measuring dietary intake. Most technology assisted methods reduce the time and costs associated with data collection by automating some aspects of the data collection, coding, and analysis. Removing the face to face element of traditional methods may also allow for a larger sample of individuals to be assessed. However, this application of technology does not usually overcome the measurement biases commonly associated with assessing dietary intake. Technology is also being used to develop new methods which may address some of these biases, such as using photography to capture intake instead of self-report [1], or physiological measurements as a proxy to intake [2]. However, these applications are generally not ready for use in large population studies.

There are some examples of where traditional methods have been transferred online and made available to researchers and the general public to assess dietary intake. One example is the Automated Self-Administered 24-h recall (ASA-24), which is an automated, self-administered 24-h recall available in the USA [3], and is currently being adapted and validated for use in the Australian population. The online platform removes the role of an interviewer in a 24-h recall, and the system provides prompts and cues to assist individuals’ in reporting all food and beverages consumed on a day, or across multiple days. By tradition, the primary output from these recalls is nutrient intake, although more recently with the use of an online platform and advances in statistical methods, multiple recalls are being administered more frequently and used to estimate usual food consumption [4].

Food frequency questionnaires (FFQs) also collect information about usual or habitual intake of a selection of foods. They are often designed to be self-administered, accommodate larger samples, and are well suited to an online delivery. The length of FFQs can vary depending on the researcher’s focus. Longer FFQs aim to capture the total food and nutrient intake but shorter versions tend only to characterise intakes of one, or a few, nutrients or food groups. The validation of FFQs has traditionally been in relation to energy and nutrient intakes but not food intake [5,6]. A recent review of short FFQs and questions to measure food intake in children and adolescents identified 30 tools that had undergone scientific validation. Only six of these short tools measured a wide range of food groups [7]. The challenge remains whether short questionnaires can be used to capture intake of a range of foods, total diet, diet quality, and/or whole dietary patterns.

The Australian Dietary Guidelines provide information on the types and amounts of food to eat from a range of food groups, and a dietary pattern to promote health and wellbeing. In recent years, the interest in methods to assess whole diet and eating patterns has increased as a way to monitor population intakes, and compare population intakes against such food based dietary guidelines. The Short Food Survey, is a short questionnaire which assesses intake of all five core food groups, beverages, discretionary choices, food choices, and variety, allowing for compliance with the Australian Dietary Guidelines to be estimated as a diet quality score [8]. The survey has been validated against 24 h recalls in adults [9] and children [10], and found to provide a reliable and adequate estimate of overall diet quality. Given the lack of scientifically developed tools available online [7], there was an opportunity to provide the Short Food Survey to Australians to assess their own dietary intake, and for researchers to find out how Australians’ diets comply with dietary guidelines. Conducting national nutrition surveys is a big financial undertaking, and as such it was almost 20 years between surveys in Australian adults [11,12]. We envisaged that a freely available tool would attract a large audience to complete the survey, and have the potential to provide a unique and contemporary insight into what Australians are eating. This information would be most useful to fill the gap in the intervals between national surveys. In May 2015, the Commonwealth Scientific and Industrial Research Organisation (CSIRO) Healthy Diet Score survey was launched online. This paper describes the reach and user experience of the survey, and summarises how compliant Australians self-reported diets are with the Australian Dietary Guidelines.

## 2. Materials and Methods

The CSIRO Healthy Diet Score was developed in partnership by the Commonwealth Scientific and Industrial Research Organisation (CSIRO) and SP Health Co. (Sydney, Australia)—an Australian business who deliver online health solutions. The CSIRO Healthy Diet Score survey is an extension of the Short Food Survey which was developed by CSIRO and the University of South Australia [9,10]. The Short Food Survey is a series of 38 short questions which were purposefully compiled to allow for the calculation of a Dietary Guideline Index score [13], referred to here as “Diet Score”. The Short Food Survey has undergone validation in a sample of Australian adults [9] and separately for parents reporting children’s intake [10], and been shown to provide a valid estimate of overall diet quality.

The Australian Dietary Guidelines provide Australians with specific advice on the quantities of core and discretionary (noncore) food and beverages to consume on a daily basis, based on their age and gender [14]. To reflect these guidelines as best as possible, the Diet Score scoring system assesses the quantity, quality, and variety of foods consumed. A detailed description of the questions and scoring algorithm are published elsewhere [9,10,13]. Briefly, for the quantity components individuals report their usual intake, in serves, per day, week or month, from which serves per day is calculated. The scoring algorithm compares the daily amount of fruit, vegetables, breads and cereals, meat and alternatives, dairy and dairy substitutes, and discretionary foods to age and gender specific recommendations. For the quality components, the frequency of wholegrains consumption, reduced fat dairy consumption, frequency of trimming meat, fat type of spreads used, and water consumption (as a proportion of total fluids) are assessed. The variety of foods consumed within each core food group is also scored. Each component of diet quality is calculated as a score out of 10, except for discretionary foods which is out of 20 points. Achievement of a maximum score indicates that an individual’s intake met the recommendation (i.e., had an optimal intake). Minimum scores were generally assigned to zero intakes, and in between scores were allocated as a proportion of the recommendation. All components are summed to give an overall Diet Score out of 100, where an individual’s Diet Score reflects their overall compliance with the Australian Dietary Guidelines, and a higher score reflects greater compliance with guidelines and higher diet quality. To aid interpretation and comparison of scores between these components, we have expressed the component scores (originally out of 10, except for discretionary foods which was out of 20) as a score out of 100 in the tables and description of results.

**Figure 1:**
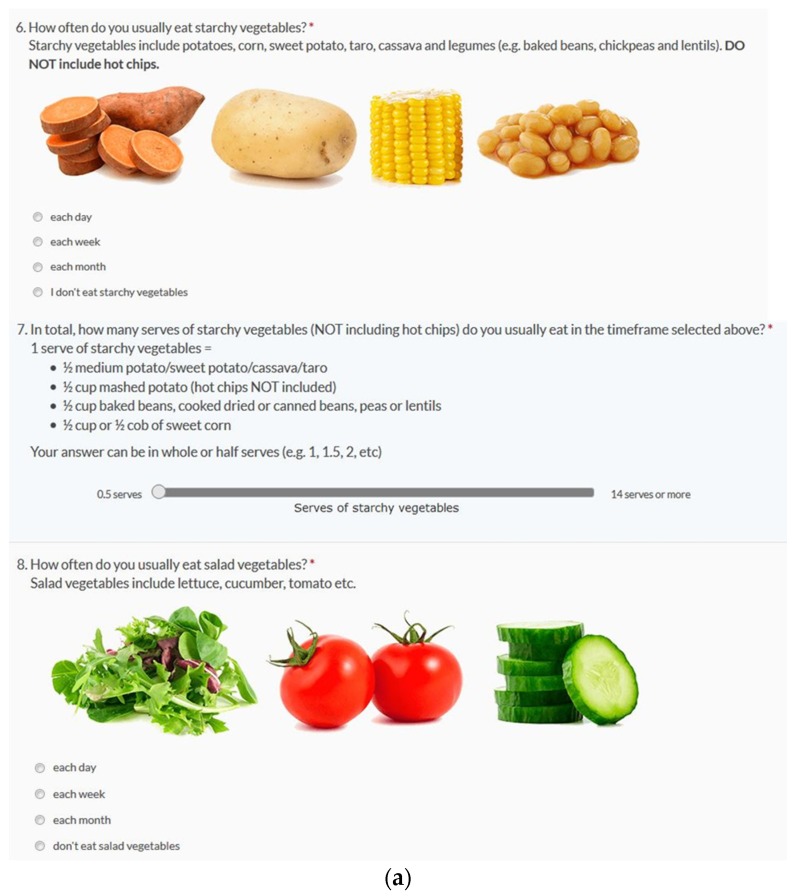

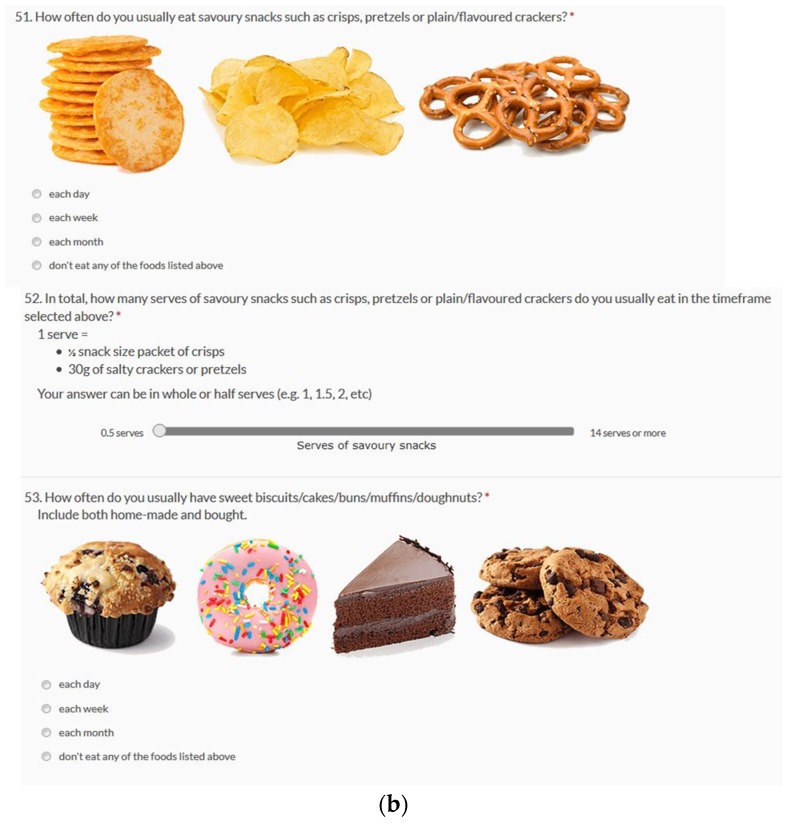
Screenshots of questions from the Commonwealth Scientific and Industrial Research Organisation (CSIRO) Healthy Diet Score survey. Examples of questions about the frequency and quantity of (**a**) Core foods and (**b**) Discretionary foods.

The CSIRO Healthy Diet Score survey contains the 38 questions asking about the frequency and amounts of different foods consumed, food choices, and variety—the same as the Short Food Survey. In addition, the CSIRO Healthy Diet Score survey contained coloured images of example foods included in each question. The portion size of the images were selected to be as realistic as possible, however were not intended to be exact serve sizes in all instances (Figure 1). The survey was programed into a readily available survey platform. On completion, individuals receive an immediate Diet Score on completion of the survey, as well as three personalized suggestions for areas to improve.

The CSIRO Healthy Diet Score survey is freely available to all Australians at the website www.csirodietscore.com, and through the CSIRO (http://www.csiro.au) and Total Wellbeing Diet (https://www.totalwellbeingdiet.com/) websites.

**Figure 2:**
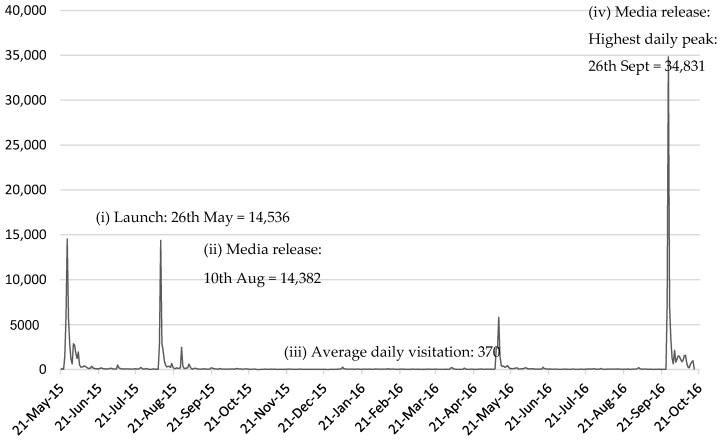
Number of people who have started the CSIRO Healthy Diet Score survey from (i) launch (21 May 2015) to current (17 October 2016) with key peaks in visitation at (ii and iv) the key media releases and (iii) average daily visitation highlighted.

**Figure 3:**
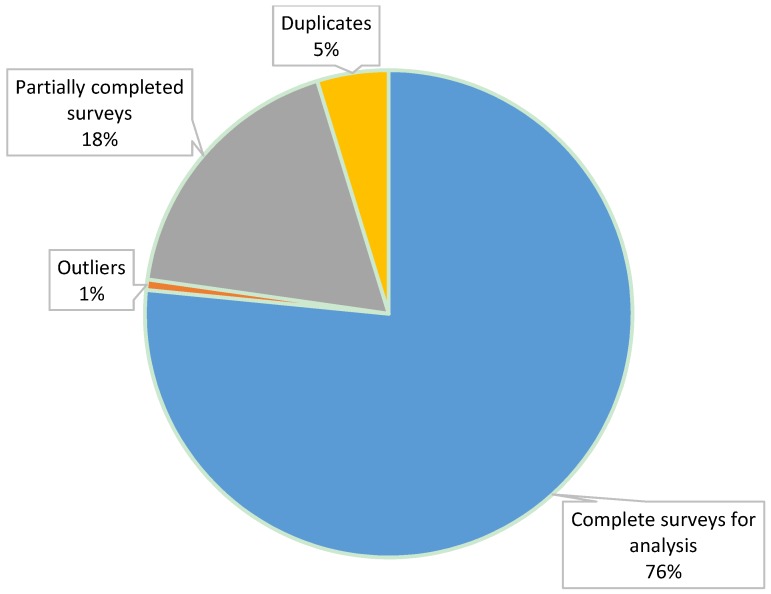
Data management and data cleaning of submitted surveys.

The CSIRO Healthy Diet Score survey is currently a live website, meaning data collection is continuous and ongoing. The survey went live on the 21 May 2015 and CSIRO and SP Health coordinated four media releases: (i) a small scale media on 21 May associated with the launch; (ii) a major release about the first results of the survey conducted on 10 August 2015; (iii) a second small scale release marking one year since launch; and (iv) a second major release of findings on 26 September 2016. The two major releases received national television, print, and radio coverage across a range of free to air television channels (e.g., news and current affairs programs), Australian print newspapers and internet stories, and local and national radio programs. The peaks in visitation to the survey corresponded with these publicity campaigns (Figure 2). This paper describes data collected from individuals who completed the survey from when it went live on the 21 May 2015 through to the 17 October 2016. During this period, the survey was commenced 190,667 times. Duplicates were identified using an ID variable (*n* = 9046) and individuals’ first attempt of completing the survey was included in the present analysis. Of the 181,621 surveys by unique users, 34,323 surveys were only partially completed, leaving 147,298 complete surveys. Outliers were identified and removed based on extreme age (less than 18 and older than 100 years removed), Body Mass Index (less than 13 and greater than 97 removed), height (less than 1m and greater than 3 m), and weight (less than 13 kg and greater than 250 kg). In total 1323 surveys were removed based on the above criteria, leaving 145,975 surveys for analysis (76.6% of those that commenced the survey, Figure 3). According to the summary statistics provided by the survey platform, the highest drop off between pages of the survey was 4% between page 2 about fruit and page 3 about vegetables. The drop between the remaining pages was 0%–2%, and there was no pattern to this drop off.

Descriptive analysis (means, standard deviations, and frequencies) were used to describe the sample included in the analysis, and their scores on each component of the Diet Score as well as the Diet Score overall. Due to the large sample size, discussion of results favoured the most meaningful differences (considered to be a difference of 5% or more between groups), as opposed to a statistically significant difference. This study was approved by the CSIRO Health and Medical Human Research Ethics Committee Low Risk Review Panel (LR 29/2016).

## 3. Results

### 3.1. Sample Characteristics

The majority of the sample was female (71%), with a relatively even distribution across the 18–30, 31–50, 51–70 years age groups (30.5%, 36.0%, 30.2% respectively, Table 1). Almost half the sample (48.2%) reported a height and weight that placed them in the normal weight category, 30.4% were overweight and 18.9% obese. The largest proportion of respondents resided in Victoria (30.8%), followed by New South Wales (27.2%) and Queensland (14.5%). There were a wide range of occupations reported by individuals. The most common occupations were management/finance (12.8%), education/research (12.4%), student (11.2%), and retired (10.6%).

### 3.2. Variation in the Diet Score within the Sample

The average Diet Score was 58.8 out of a possible 100 (SD = 12.8). Women reported a higher Diet Score than men (59.9 (SD = 12.6) vs. 56.2 (13.1)) and older adults reported a higher Diet Score than younger adults (Table 1). There was a stepwise decrease in Diet Score with increasing weight status. Normal weight adults scored 60.5 (12.6), compared to overweight and obese adults who scored 58.1 (SD = 12.5) and 55.7 (SD = 13.2), respectively (Table 1). Obese men had the lowest Diet Score (M = 52.6, SD = 13.2) and normal weight women the highest score (M = 61.3, SD = 12.3) of all gender/weight status groups.

There was little variation between Australian states, with only 2.2 points separating the highest (the Australian Capital Territory) and lowest (Tasmania) states. Over half the sample resided in New South Wales (27.2%) and Victoria (30.8%) and the average score for these states were among the highest at 59.2 (SD = 12.8) and 59.0 (SD = 12.8), respectively (Table 1).

There was significantly more variation in average Diet Score by occupation. Adults working in the health industry (M = 61.9, SD = 12.3) and those who were retired (M = 62.8, SD = 12.0) reported the highest scores; whereas construction workers (M = 54.2, SD = 13.4) and those who were unemployed (M = 54.1, SD = 15.0) reported the lowest Diet Scores.

### 3.3. How do Australian Diets Compare to the Australian Dietary Guidelines?

An average Diet Score of 58.8 (SD = 12.9), suggests that many areas of the diets of this sample were not consistent with the Australian Dietary Guidelines. The highest component score was for fluid, meaning that most people were reporting to consume more water than sugar-sweetened beverages. The average component scores for vegetables and meat and alternatives were similar (about 70 out of 100, Table 2). However, there were gender differences in these score, and in particular for vegetables and discretionary foods. The average vegetable score for women was 74.0 (SD = 27.8), which was almost 17% higher than the average male score of 61.6 (SD = 29.6). In keeping with this, women scored 20% better than men for discretionary foods, but for both groups scores were poor and lowest of all component scores (M = 25.8 for men and M = 32.0 for women). Overall, women scored higher than men on all food group components, except breads and cereals (Table 2).

### 3.4. User Feedback

At the completion of the Survey, users receive immediate feedback on the three poorest performing areas of their diet. Almost three-quarters of the sample received feedback about their overconsumption of discretionary foods, meaning for 73.8% of the population discretionary foods was in their bottom three scores (Table 3). Dairy foods (55.5% of the sample) and healthy fats (47% of the sample) were the other two most commonly received feedback messages. Receiving feedback about discretionary foods was more common in men than women (76.8% vs. 72.5%), as was feedback about under consumption of vegetables (32.8% vs. 20.6%). Women were more likely than men to receive feedback about their consumption of breads and cereals (29.6% vs. 23.0%), dairy foods and their substitutes (57.2% vs. 51.3%), healthy fats (47.7% vs. 45.3%), and variety (15.0% and 11.4%, Table 3).

## 4. Discussion

This study found that the average Diet Score for a sample of over 145,000 Australians was 59 out of 100, meaning that, at large, Australians self-reported diets are not consistent with the recommendations in the Australian Dietary Guidelines. While women scored better than men, the poorest performing areas for all adults were discretionary foods, dairy foods, and healthy fats. The overconsumption of discretionary foods is problematic for a number of reasons. Firstly, discretionary foods are energy dense, and overconsumption of these foods can lead to overconsumption of energy and weight gain [14]. Secondly, discretionary foods, which are high in saturated fat, salt, and sugar but low in essential nutrients, can displace nutrient-dense core foods—meaning the overall nutritional adequacy of the diet may be compromised.

It is well established that women have healthier dietary patterns than men [15,16], and here we found that women scored better than men on nearly all aspects of diet quality, but in particular vegetables and discretionary foods. On average women scored 17%–20% better on these two dietary components. Vegetables are a key marker of a healthy diet [17,18] and the focus of leading public health nutrition messages globally [14,19,20,21]. Discretionary foods traditionally have not received the same attention, but given they account for about 35% of total energy intake [22] and in the light of the current rates of obesity, an alternative approach to address healthy eating may be through starting from a message of eating less discretionary foods [23].

It is assumed, but not always the case in survey data, that overweight and obese individuals consume more energy and have poorer diets than adults of a normal weight. Using short questions delivered in an online format, we found overweight and obese adults reported lower Diet Scores than normal weight adults. It is unusual to see this finding in large nutrition surveys within Australia [11] and internationally [24]. The inability of traditional surveys to show increases in energy intake with weight status is often associated with potential underreporting of intake, and the likelihood that underreporting is more frequent and of a greater magnitude in obese individuals [25,26,27]. It has been estimated that Australian adults may under report their energy intake by up to 20% [11], and this is more likely due to an under reporting of unhealthy discretionary foods. Misreporting of intake can be the result of issues associated with recall—that is remembering what and how much you consumed; or social bias—that is adjusting what you report to eat because you think you might be judged [28]. The anonymity of online completion may remove some of this perceived judgement, resulting in a reported intake closer to true intake. In addition, the presence of pictures to assist the recall of foods in this survey may also have partially addressed some of misreporting, but we cannot know the extent to which each of these have helped. Also, there will still be some degree of error associated with individuals’ ability to aggregate intake into groupings as a result of the style of questioning used in this survey tool. Like all dietary assessment methods, we will continue to refine the methods used to minimize bias and develop a tool which estimates food intake as accurately as is possible.

The context in which one lives and works can impact on health and health outcomes [29,30]. While we found little variation in Diet Score by Australian state of residence, there were interesting patterns in Diet Score by occupation. Adults working in the health industry and those who were retired scored between 62 and 63 out of 100, compared to construction workers and those who were unemployed who score 54 out of 100—which was 5 points lower than the Australian average. Occupation, like income, has been used as a proxy for socioeconomic status, and poorer dietary intake and health outcomes [31] have been associated with lower socioeconomic status. Variation in nutrition knowledge, beliefs and food skills might also differ between groups of different occupations, which may also explain some of these differences in intake. Tailored workplace based nutrition interventions can address some of the challenges faced by people in different workplaces such as access to healthy food on site or on the road and are largely unexplored. These may be an effective approach to reaching population groups who are known to be at greater risk of poor intake.

The media releases for the Survey received national coverage, and attracted a lot of traffic to the website, resulting in Australia’s largest ever survey of dietary intake. The sample analysed here was about 15 times larger than the National Nutrition Survey conducted as part of the 2011–2012 Australian Health Survey [15], however the media attention was not able to attract a nationally representative population—which is a key feature of the sophisticated sampling techniques used in national surveys [11]. The National Nutrition Survey also applies a population weighting to data to allow statements about the intake of the Australian population to be made. There is potential to apply population weighting to CSIRO Healthy Diet Score survey sample in future analyses. As is commonly observed with health related surveys, the majority of the current sample were women (71% compared to 51% within the Australian population) [32]. In addition, our sample had a higher proportion of young adults than the Australian population (18–30 years: ~30% vs. ~19% nationally) [32]. Normal weight adults made up the greatest proportion of our sample, but still almost 50% of adults in this survey were overweight or obese (compared to 63% nationally) [15]. While there were a range of occupations represented in our sample, the distribution may have also influenced the overall results.

Given our finding that obese adults had poorer diet quality than normal weight adults, and the overweight and obese adults were underrepresented relative to national estimates, it is likely that the overall Diet Score of all Australians would actually be lower than what has been reported here. Regardless, we found that at a national level compliance with dietary guidelines is poor. To close this gap, there is a need for nutrition promotion, practical support, and even tailoring of nutrition education initiative to specific groups to improve the overall dietary intake of the Australian population.

This project also demonstrates that readily available survey tools are suitable for use when collecting data from large samples and their automated downloads make it easy to extract large datasets. Interestingly, despite the advances in technology and the ubiquitous nature of computers and electronic devices, a recent review of short dietary assessment tools found only four tools were administered via a computer—the majority were still administered in a paper format [7]. A second advantage of the online environment is the cost effectiveness of scalability. Once established, there is little to no additional cost associated with collecting large amounts of data. Conversely, traditional methods of dietary assessment are resource intensive and each administration generally takes time, which equates to money. Data cleaning becomes an important process for large, online surveys. We removed partially completed surveys; duplicate administrations for the same user; and excluded surveys based on erroneous age, BMI, height, and weight values. Time associated with data analysis is generally constant regardless of the amount of data collected, so overall online delivery of dietary assessment tools is a cost effectiveness mechanism.

The online environment was an asset in this study, allowing individuals to receive their Diet Score and personalized feedback immediately on completion of the survey. This type of feedback, at a food group level, is not always possible with traditional approaches to dietary assessment or even with short FFQ type surveys delivered in traditional ways. We are unsure of how this feedback changed dietary behavior, however this is an opportunity for future research. Another area for future research is to define a ‘good’ score. The average Diet Score of the highest quintile (top 20%) of this sample was about 75, which might be a benchmark for a ‘good’ score, but we cannot substantiate this with health outcomes, other that weight status. A recent examination of diet quality using the Australian Health Survey data reported higher diet quality was associated with lower odds ratio of hypertension in Australian men but not women [16]. More longitudinal research is need to establish whether diet quality, in this case Diet Score, predicts health and risk of disease.

There are other Australian diet quality indexes which have been developed primarily for research purposes that can be applied to FFQ [33] and 24-h recall data [8] or state-based health surveys [34]. There are also two Australian online FFQs which have been validated to provide estimates of usual nutrient intake [35,36,37,38], and one of these also offers immediate analysis of results and comparisons with food-based recommendations [37]. However there is a cost associated with using both of these online surveys, and only one, the Australian Eating Score, is available to individuals interested in evaluating their own diet [37]. The advantage of making the Diet Score survey freely available is that we have reached an unprecedented number of individuals, as well as a national audience. However, this may also be considered a limitation as it attracts motivated, health interested individuals. Regardless, this survey has provided some interesting insights about the food intake of many Australians, and will continue to provide these insights on a more regular basis than the National health and nutrition surveys are able to.

## 5. Conclusions

This paper describes the implementation and delivery of an online survey to estimate compliance with the Australian Dietary Guidelines through the CSIRO Healthy Diet Score. In approximately 18 months, 145,000 Australians have completed the survey and provided valuable insights into the food intake of the Australian population. The use of technology has enhanced the reach and also the user experience of completing a dietary assessment tool, where users receive an immediate Diet Score and feedback on three areas they could improve. This tool has the potential to increase individuals’ awareness of their own diet quality, and quantify the disconnect between what they eat and what the recommendations suggest they should eat. Over time, as more Australians complete the survey, the data will be useful to monitor population diet quality and understand the degree to which the population’s diet complies with the Australian Dietary Guidelines.

## Figures and Tables

**Table 1:** Characteristics of the CSIRO Healthy Diet Score Survey Sample and Summary Diet Scores by Demographic Characteristic (*n* = 145,975).

		Sample Characteristics		Diet Score	
		Count (*n*)	% of Total	Mean	SD
Gender	Male	42,385	29.0%	56.2	13.1
	Female	103,590	71.0%	59.9	12.6
Age group	18–30 years	44,534	30.5%	57.3	13.2
	31–50 years	52,599	36.0%	57.3	12.6
	51–70 years	44,096	30.2%	61.8	12.2
	71+ years	4746	3.3%	63.1	11.7
Weight status	Underweight	3685	2.5%	59.6	14.4
	Normal weight	70,205	48.2%	60.5	12.6
	Overweight	44,376	30.4%	58.1	12.5
	Obese	27,517	18.9%	55.7	13.2
State of residence	New South Wales	39,313	27.2%	59.2	12.8
	Queensland	20,988	14.5%	58.2	13.1
	Australian Capital Territory	6047	4.2%	59.7	12.4
	Northern Territory	1197	0.8%	58.3	12.7
	Tasmania	4418	3.1%	57.6	13.1
	Victoria	44,558	30.8%	59.0	12.8
	Western Australia	13,415	9.3%	58.2	12.6
	South Australia	14,631	10.1%	58.7	12.9
Occupation	Retired	15,238	10.6%	62.8	12.0
	Administration	13,645	9.5%	57.8	12.7
	Student	16,152	11.2%	58.4	13.2
	Health industry	13,799	9.6%	61.9	12.3
	Education/Research	17,896	12.4%	59.9	12.1
	Science/Programming	8728	6.1%	57.2	12.6
	Homemaker	6398	4.4%	59.1	13.0
	Management/Finance	18,394	12.8%	58.0	12.3
	Sales/Marketing/PR	7723	5.4%	56.4	12.8
	Customer and Food Service	6028	4.2%	55.4	13.8
	Media/Arts	3187	2.2%	58.2	12.3
	Construction Industry	2860	2.0%	54.2	13.4
	Unemployed	2118	1.5%	54.1	15.0
	Other	11,807	8.2%	58.0	13.1

**Table 2:** CSIRO Healthy Diet Score and Food Group Components Scores by Gender and for the Total Sample (*n* = 145,975).

Component Scores (out of 100) *	Male		Female		Total	
	Mean	SD	Mean	SD	Mean	SD
Diet Score	56.2	13.1	59.9	12.6	58.8	12.9
Food Group Component Scores						
Fluids	88.7	18.4	93.6	14.0	92.2	15.5
Vegetables	61.6	29.6	74.0	27.8	70.4	28.9
Meat and alternatives	68.3	26.1	70.9	25.3	70.2	25.6
Fruit	65.9	36.8	68.7	35.2	67.9	35.7
Variety	64.4	13.3	65.0	13.0	64.9	13.1
Breads and cereals	62.2	24.6	60.9	24.4	61.3	24.4
Healthy fats	50.9	28.7	54.1	26.7	53.1	27.3
Dairy and substitutes	47.8	25.5	48.0	26.5	47.9	26.2
Discretionary foods	25.8	31.0	32.0	32.3	30.2	32.1

* Component scores are expressed as a score out of 100 for ease of comparison between components.

**Table 3:** Frequency of User Feedback by Food Group and Gender (*n* = 145,975).

Food Group Component	Male	Female	Total
	%	%	%
Discretionary foods	76.8	72.5	73.8
Dairy and substitutes	51.3	57.2	55.5
Healthy fats	45.3	47.7	47.0
Fruit	33.1	32.5	32.7
Breads and cereals	23.0	29.6	27.7
Vegetables	32.8	20.6	24.2
Meat and alternatives	22.4	22.6	22.7
Variety	11.4	15.0	14.0
Fluids	3.9	1.9	2.5
